# Improvement of Antioxidant Defences in Keratinocytes Grown in Physioxia: Comparison of 2D and 3D Models

**DOI:** 10.1155/2023/6829931

**Published:** 2023-06-17

**Authors:** Nadira Chettouh-Hammas, Fabienne Fasani, Amandine Boileau, David Gosset, Giovanni Busco, Catherine Grillon

**Affiliations:** Center for Molecular Biophysics UPR4301 CNRS, Rue Charles Sadron, 45071 Orléans Cedex 2, France

## Abstract

Keratinocytes prevent skin photoaging by ensuring the defence against oxidative stress, an excessive production of reactive oxygen species (ROS). They are localized within the epidermis where the oxygen level (1-3% O_2_), named physioxia, is low compared to other organs. Oxygen is essential for life but also generates ROS. Most of the *in vitro* studies on keratinocyte antioxidant capacities are performed under atmospheric oxygen, named normoxia, which is very far from the physiological microenvironment, thus submitting cells to an overoxygenation. The present study is aimed at investigating the antioxidant status of keratinocyte grown under physioxia in both 2D and 3D models. First, we show that the basal antioxidant profiles of keratinocytes display important differences when comparing the HaCaT cell line, primary keratinocytes (NHEK), reconstructed epidermis (RHE), and skin explants. Physioxia was shown to promote a strong proliferation of keratinocytes in monolayers and in RHE, resulting in a thinner epidermis likely due to a slowdown in cell differentiation. Interestingly, cells in physioxia exhibited a lower ROS production upon stress, suggesting a better protection against oxidative stress. To understand this effect, we studied the antioxidant enzymes and reported a lower or equivalent level of mRNA for all enzymes in physioxia conditions compared to normoxia, but a higher activity for catalase and superoxide dismutases, whatever the culture model. The unchanged catalase amount, in NHEK and RHE, suggests an overactivation of the enzyme in physioxia, whereas the higher amount of SOD2 can explain the strong activity. Taken together, our results demonstrate the role of oxygen in the regulation of the antioxidant defences in keratinocytes, topic of particular importance for studying skin aging. Additionally, the present work points out the interest of the choice of both the keratinocyte culture model and the oxygen level to be as close as possible to the *in situ* skin.

## 1. Introduction

Oxygen is one of the main parameters of the microenvironment in tissues where it ensures an optimal cell metabolism required for keeping homoeostasis in physiological conditions. In the skin, the physiological oxygen level (physioxia) ranges from 1 to 3% in the epidermis, and 5 to 7% in the dermis, and is lower as compared to the atmospheric oxygen, 21% (normoxia) [1]. However, *in vitro* cell culture experiments are commonly performed under standard atmospheric oxygen tension reaching around 18.6% O_2_ inside an incubator [2], which is far from the physiological oxygen level. Several studies have demonstrated the effect of an overoxygenation on cell activities and shown the importance of maintaining the physioxia of each tissue in *in vitro* assays. As an example, keratinocytes grown under elevated oxygen levels were shown to consume more oxygen and to produce higher ATP levels [3]. Indeed, the variation in oxygen can impact the cell metabolism [4], proliferation and differentiation [5], and key mechanisms of skin functions.

A consequence of oxygen metabolism in cells is the production of reactive oxygen species (ROS), and their amount depends on the environmental oxygen availability [5–9]. A basal intracellular ROS level is required for cell life and physiological functions as these species are involved in cell proliferation and differentiation, in vascular tone and apoptosis regulation, and also in the defence against microorganisms [10]. However, an excess of ROS can be deleterious. In particular, keratinocytes are the main targets of several exposome factors such as UV radiations, air pollutants, and stress [11], which lead to the generation of high ROS levels and to oxidative stress. The latter causes DNA mutations as well as proteins and lipid oxidations inducing apoptosis [12] and keratinocyte senescence [13]. All these events lead to the acceleration of skin aging and potentially to pathologies.

Therefore, cells developed various strategies to convert ROS into less toxic species, thus preventing their accumulation and the subsequent potential damages. The first effective line of defence against ROS involves antioxidant enzymes, mainly the superoxide dismutases (SODs) responsible for the neutralization of O_2_^•-^ to H_2_O_2_, which is then eliminated by catalase or glutathione peroxidase (GPX) metalloenzymes. These enzymes have distinct and specific mechanisms of action and different subcellular localizations. However, they can act in a complementary way in order to avoid oxidative damages to the cellular components. In the skin, these enzymes are more expressed in the epidermal layer than in the dermis [14], where they protect the tissue and thus prevent skin photoaging. Their inefficiency negatively impacts the skin homoeostasis [15].

In the current work, we investigated the role of physiological oxygen level in the modulation of the antioxidant system in keratinocytes, both in monolayer cultures and in reconstructed epidermis. First, we compared the antioxidant enzyme profile between the 2D (HaCaT cell line and primary keratinocytes) and 3D keratinocyte models. We then established and characterized the *in vitro* models in physioxia versus normoxia conditions and compare their redox state by evaluating ROS production. To better understand the observed modulations, we studied catalase, superoxide dismutases, and glutathione peroxidases at the level of gene expression and protein amount as well as their antioxidant activity.

## 2. Materials and Methods

### 2.1. Monolayer Cell Cultures

HaCaT cell line was derived from the spontaneous immortalization of human keratinocytes [16] and was purchased from CLS GmbH (Eppelheim, Germany). HaCaT cells were maintained in Dulbecco's Modified Eagle's Medium (DMEM) (Gibco™, Thermo Fisher Scientific, Illkirch-Graffenstaden) supplemented with 10% fetal bovine serum (Sigma-Aldrich, Saint-Quentin-Fallavier, France), 100 U/mL penicillin, 100 *μ*g/mL streptomycin (Gibco™, Thermo Fisher Scientific), and 2 mM L-glutamine (Eurobio, Les Ulis, France). The primary normal human epidermal keratinocytes (NHEK) were purchased from Lonza Bioscience (Basel, Switzerland) and maintained in DermaLife K complete medium (Lifeline Cell Technology, Frederick, MD, USA) supplemented with 100 U/mL penicillin and 100 *μ*g/mL streptomycin. Normal human adult primary fibroblasts (HDF) were purchased from Cell Systems (Kirkland, WA, USA) and maintained in FibroLife medium (Lifeline Cell Technology) supplemented with 100 U/mL penicillin and 100 *μ*g/mL streptomycin. Cultures were grown at 37°C in normoxia condition in a classical incubator with 5% CO_2_ and 18.6% of O_2_, or in physioxia condition inside a HypOxystation® H35 (Don Whitley, Bingley, United Kingdom) with 3% of O_2_, 5% CO_2_, and 92% N_2_. Throughout this study, cells were always used at about 70% confluence with a maximum of 40 passages for HaCaT cell line, of 2-3 passages for NHEK, and of 6 passages for HDF. The numbers of viable cells were determined using the NucleoCounter (ChemoMetec, Allerod, Denmark) according to the manufacturer's instructions.

### 2.2. Reconstructed Human Epidermis

Reconstructed human epidermis (RHE) were generated on polycarbonate membrane cell culture inserts with 12 mm diameter and 0.4 *μ*m pore size (Sigma). Briefly, after thawing, HDF and NHEK were resuspended in their specific culture medium and maintained in normoxia or in physioxia conditions, the medium being replaced after 24 hours and every two days. When cells reach 70% confluence, HDF were harvested by incubation with trypsin-EDTA (Gibco™, Thermo Fisher Scientific), seeded at 5 × 10^4^ cells/insert on the outer side of the insert membrane, and let attach for 1 h. Keratinocytes were harvested using trypsin solution (Lonza Biosciences) and seeded into the inserts at 4 × 10^5^ cells/insert in DermaLife K complete medium. Inserts were placed in 6-well plates containing 2 mL DermaLife K complete medium and incubated for 48 h. Then, to induce keratinocyte differentiation, the medium inside the insert was removed and keratinocytes were subsequently exposed to the air-liquid interface. The medium inside the 6-well plates was replaced by 1.5 mL of EpiLife supplemented with 1% Human Keratinocyte Growth Supplement (Gibco™, Thermo Fisher Scientific), 1.5 mM CaCl_2_, 92 *μ*g/mL L-ascorbic acid 2-phosphate, and 10 ng/mL keratinocyte growth factor (Sigma-Aldrich). The medium was renewed every 2 days, and the air-liquid cultures were maintained for 15 days.

### 2.3. Skin Explants

Abdominal skin biopsy from a 37-year-old woman was obtained from Proviskin (Besançon, France). Explants of 1 cm diameter were prepared from defatted skin biopsy, incubated in RNAlater solution (Invitrogen, Thermo Fisher Scientific) for 24 h at 4°C, and immediately frozen and kept at -80°C before RNA extraction.

### 2.4. Measurement of Intracellular ROS Generation

Intracellular ROS formation in normoxia and physioxia was measured by the cell-permeant probe 5-(and-6)-chloromethyl-2′,7′-dichlorodihydrofluorescein diacetate (CM-H_2_DCFDA, Invitrogen™, Thermo Fisher Scientific). Cells were seeded at a density of 1 × 10^3^/well in 96-well microplates and were maintained for four days in normoxia or physioxia condition. Then, cells were washed with phosphate-buffered saline (PBS) and incubated with 10 *μ*M CM-H_2_DCFDA for 20 min. After washings, cells were stressed with 50, 100, 200, or 400 *μ*M H_2_O_2_ (Sigma-Aldrich) for 20 min. We previously checked that the 20 min incubation with the highest concentration of H_2_O_2_ does not lead to cell toxicity (88 to 94% cell viability). Nonstressed cells were incubated with N-acetyl-cysteine 5 mM (NAC, Sigma-Aldrich), a ROS scavenger, during the previous two steps. The relative fluorescence intensity was then measured using a microplate spectrophotometer (VICTOR 3V, Perkin Elmer, Villebon-sur-Yvette, France) with excitation at 490 nm and emission at 535 nm. The results are expressed in relative fluorescence units for the nontreated cells and in fold change versus control for H_2_O_2_-treated samples.

### 2.5. Western Blots

After culture, cells were washed with cold PBS and then mechanically harvested in RIPA lysis buffer (Santa Cruz Biotechnology, Dallas, TX, USA). After a 15 min incubation on ice, cell lysates were centrifuged at 10,000 × g for 15 min. After collection of supernatants, protein extract concentration was determined using Bio-Rad DC™ protein assay (Bio-Rad, Hercules, CA, USA). Proteins were denatured at 95°C for 5 min in the Laemmli buffer with 0.1% *β*-mercaptoethanol (Bio-Rad). 25 *μ*g of proteins were then loaded on a 12% SDS-PAGE gel at a voltage of 160 V for 45 min. The separated proteins were transferred to a nitrocellulose membrane (Bio-Rad) using Trans-Blot Transfer System (Bio-Rad). Membrane was incubated in a blocking buffer (5% dry skimmed milk in Tris-buffered saline (TBS) supplemented with 0.1% Tween-20). Proteins were probed with primary antibodies overnight at 4°C and then with horseradish peroxidase-conjugated secondary antibodies for 1 h at room temperature. After washing with TBS-Tween-20, bands were detected with enhanced chemiluminescence reagents (West Dura detection kit, Thermo Fisher Scientific), and then, the signal was digitally acquired using the chemiluminescence imager ChemiDoc (Bio-Rad). The following primary antibodies were used: catalase (1 : 1000, 12980, Cell Signaling Technology, Danvers, MA, USA), SOD1 (1 : 1000, ab183881, Abcam, Cambridge, United Kingdom), SOD2 (1 : 1000, ab13533, Abcam), GPX4 (1 : 1000, ab13533, Abcam), and *β*-actin (1 : 10000, 4967, Cell Signaling Technology), and the secondary antibody used was goat anti-rabbit (1 : 30000, 65-6120, Invitrogen, Thermo Fisher Scientific). Protein quantification was performed using ImageLab software (Bio-Rad), and the results were normalized to the corresponding actin content.

### 2.6. Gene Expression

Total RNA from cell cultures, RHE samples, or skin explants was extracted using Qiagen miRNeasy mini kit following the manufacturer's recommended protocol (Qiagen, Hilden, Germany). The reverse transcription of 1 *μ*g total RNA was performed using reverse transcription kit Maxima First Strand cDNA (Fermentas, Thermo Fisher Scientific). The generated cDNA (200 ng) was used as a template for the qPCR with various primers and SYBR Premix Ex Taq^TM^ (TAKARA BIO INC., Saint-Germain-en-Laye, France). PCR was set up in duplicate. A slight modification of the manufacturer's protocol allows the use of 2 *μ*L cDNA per reaction, instead of 5 *μ*L. Reactions were run on a LightCycler 480 (Roche Diagnostics, Meylan, France). A dissociation curve was run after each PCR reaction to ensure that only one amplicon was synthetized. qPCR analysis was performed using the LightCycler 480 software. The efficiency of each primer pair was calculated using a dilution range of cDNA. The relative quantity of each gene was then calculated by considering the efficiency of each primer pair. Three housekeeping genes, glyceraldehyde-3-phosphate dehydrogenase (GAPDH), beta-2 microglobulin (B2M), and *β*-glucuronidase (GUSB), were used in the same experiment. The geometric mean of reference gene-relative quantities was used to normalize the relative quantities of each gene of interest. The results are expressed as mean ± SEM of normalized relative quantities from at least three independent samples. The following primers were used in the study of the respective target genes: catalase (Sigma, FH3_CAT GGACTTTTTACATCCAGGTC and RH3_CAT CGGTTTAAGACCAGTTTACC), SOD1 (Sigma, FH1_SOD1 GAGCAGAAGGAAAGTAATGG and RH1_SOD1 GATTAAAGTGAGGACCTGC), SOD2 (Sigma, FH1_SOD2 ATCATACCCTAATGATCCCAG and RH1_SOD2 AGGACCTTATAGGGTTTTCAG), GPX1 (Qiagen QT00203392), GPX4 (Qiagen, QT00067165), B2M (Qiagen, QT00088935), GAPDH (Qiagen, QT00079247), and GUSB (Qiagen, QT00046046).

### 2.7. Measurement of SOD and Catalase Enzymatic Activities

After four days of culture in normoxia or physioxia, the antioxidant activities in HaCaT, NHEK, and RHE were measured using catalase and SOD assay kits (707002 and 706002, Cayman Chemicals, Ann Arbor, MI, USA). Briefly, cells were harvested in the corresponding lysis buffer and centrifuged at 12,000 × g for 15 min and 5 min at 300 × g for catalase and SOD assays, respectively. The supernatant was kept on ice and used for the activity assay and protein quantification. The assays were performed according to the manufacturer's instructions. To measure catalase activity, methanol is converted to formaldehyde in the presence of H_2_O_2_. Then, the reaction of formaldehyde with 4-amino-3-hydrazino-5-mercapto-1,2,4-triazole (Purpald) generated a colored compound which absorbs at 520 nm. The catalase unit was described as the quantity of enzymes that degraded 1 nmol of H_2_O_2_ to O_2_ and H_2_O per minute. For SOD activity measurement, the reaction is based on a xanthine oxidase system, which produces O_2_^•−^ in the environment and then oxidizes tetrazolium into colored compounds (formazan). In this case, SODs compete with tetrazolium and lead to a decrease in coloration, proportional to the level of SOD activity. The absorbance was measured at 450 nm, and the SOD unit is defined as the amount of enzyme needed to exhibit 50% dismutation of O_2_^•−^. The results are expressed as units of enzyme/mg proteins.

### 2.8. Cell Immunostaining

Cells were seeded on glass coverslips and maintained in culture in normoxia or physioxia. They were washed with TBS and then fixed for 7 min with 4% paraformaldehyde solution on ice. Plasma membranes were permeabilized for 10 min with TBS-Triton X-100 1%, and nonspecific binding sites were saturated for 1 hour at 4°C with TBS containing 1% bovine serum albumin, 3% goat serum, and 0.3% Triton X-100. Cells were then incubated overnight at 4°C with primary antibodies. After 3 washings with TBS, cells were incubated for 1 hour with secondary antibodies. Before mounting, nuclei were stained with a solution of 50 *μ*g/mL bisbenzimide (Sigma-Aldrich) for 10 min at room temperature. Finally, coverslips were mounted on glass slides using Fluoromount-G mounting medium (SouthernBiotech, Birmingham, AL, USA). The following primary antibodies were used: catalase (12980, Cell Signaling), SOD1 (ab183881, Abcam), SOD2 (ab13533, Abcam), GPX4 (ab13533, Abcam), GPX1 (3286, Cell Signaling), and Alexa Fluor 568-conjugated goat anti-rabbit was used as the secondary antibody (A-11036, Invitrogen, Thermo Fisher Scientific).

### 2.9. RHE Immunostaining

After 15 days of culture, RHE were fixed with a 4% paraformaldehyde solution for 24 h and kept in 30% sucrose for 48 h at 4°C. RHE on membranes were separated from the insert and embedded in OCT embedding matrix (Leica, Wetzlar, Germany). Immediately, specimens were snap-frozen in isopentane on dry ice and then kept at -20°C. RHE were cut into 7 *μ*m thick slices with a cryomicrotome (Leica). Before immunostaining, cryosections were incubated in citrate buffer (0.01 M and pH 6) at 90°C for 45 min and saturated with TBS containing 1% bovine serum albumin, 3% goat serum, and 0.3% Triton X-100, for 1 hour. The immunohistofluorescent staining was performed using primary antibodies specific to Ki67 (550609, BD Pharmingen, Le Pont-de-Claix, France), involucrin (I9018, Sigma-Aldrich), loricrin (PRB-145P, Covance ref.), catalase (12980, Cell Signaling), SOD1 (ab183881, Abcam), and SOD2 (ab13533, Abcam) which were incubated overnight at 4°C. Cryosections were subsequently incubated with the corresponding secondary antibodies (Alexa Fluor 568 goat anti-rabbit IgG, A-21069, or Alexa Fluor 488 goat anti-mouse IgG, A-21121, Invitrogen) for 1 h at room temperature. Before mounting, nuclei were stained with a solution of 50 *μ*M bisbenzimide (Sigma-Aldrich) for 10 min at room temperature. Finally, cryosections were mounted using Fluoromount-G mounting medium (Thermo Fisher Scientific).

### 2.10. Immunostaining Analysis

Immunostaining images were taken using a confocal microscope LSM980 AS2 (Zeiss, Jena, Germany) and ZEN software (Zeiss). Quantification of fluorescence intensity was determined using ImageJ software and normalized to the analysed surface. For Ki67 labelling, the percentage of positive cells was assessed by the ratio between the number of Ki67-positive cells divided by the total number of nuclei (bisbenzimide positive cells) in the basal layer.

### 2.11. Statistical Analysis

All experiments were repeated at least three times as mentioned in the figure legends. The data were analysed and the figures were generated with Prism GraphPad 9 software (GraphPad, Inc., La Jolla, CA, USA). The results are presented as mean ± SEM of at least three biological replicates. For the statistical analyses, an unpaired Mann-Whitney test was used to compare two independent groups and a two-way analysis of variance (ANOVA) followed by Tukey's post hoc correction was performed for multiple comparison. The differences were considered statistically significant when ^∗^*p* < 0.05, ^∗∗^*p* < 0.01, ^∗∗∗^*p* < 0.001, and ^∗∗∗∗^*p* < 0.0001, respectively.

## 3. Results and Discussion

The role of oxygen in skin physiology is complex and difficult to study as the skin microenvironment, and mainly the physiological oxygen level, physioxia, is far from the *in vitro* culture conditions which are mostly performed under atmospheric oxygen pressure. In the present study, we used and compared several models, from keratinocyte cultures in monolayers (cell line and primary keratinocytes) to reconstructed epidermis, to investigate the status of the antioxidant system in keratinocytes in the physiological conditions of skin oxygenation.

### 3.1. Keratinocytes Express Different Antioxidant Profiles between Monolayer Cultures and Reconstructed Epidermis

To go deeper in the knowledge of antioxidant properties of skin cells, we started by comparing the expression of antioxidant enzymes in various models commonly used to study skin activities. The HaCaT cell line resulting from a spontaneous immortalization of epidermal cells is widely used as a model of keratinocytes although it is not able to achieve a total differentiation [16]. Primary keratinocytes, NHEK cells, are the best model to study epidermis activity as they are freshly isolated from the skin and closer to physiology compared to a cell line. But, under monolayer culture, both HaCaT and NHEK do not reproduce the natural multilayer architecture of the epidermis. However, these two cell culture models are the most commonly used to assess response to oxidative stress and to evaluate the activity of potential antioxidant compounds. To recreate a representative skin barrier, reconstructed human epidermis, RHE, from primary keratinocytes bring a good alternative to skin explants and is more and more studied. Beyond their three-dimensional structure, RHE develop a *stratum corneum* comparable to that observed in real skin even if its permeability is higher [17, 18].

**Figure 1:**
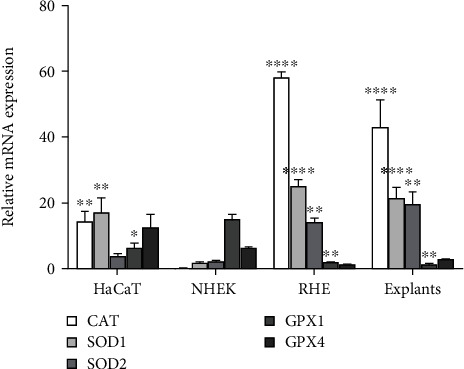
The antioxidant expression profiles are different between HaCaT, NHEK, RHE, and skin explants. HaCaT cells, NHEK, and RHE in normoxia and skin explants were analysed for the expression of mRNA of antioxidant enzymes. The results are expressed as normalized relative mRNA expression (mean ± SEM) for catalase, superoxide dismutases 1 and 2, and glutathione peroxidases 1 and 4 (*N* = 3 for HaCaT, NHEK, and explants and *N* = 6 for RHE). Statistical significance was determined using analysis of variance (ANOVA), comparing each mRNA expression in NHEK to their expression in other models. ^∗^*p* < 0.05, ^∗∗^*p* < 0.01, ^∗∗∗^*p* < 0.001, and ^∗∗∗∗^*p* < 0.0001. There is no statistical difference for each mRNA expression when comparing RHE and skin explant.

The antioxidant gene expression profile of keratinocytes grown in these models in normoxia was analysed using RT-qPCR and compared to that of skin explants. The results show clear and significant differences in enzyme expression (Figure 1). The major difference is that cells in monolayers express globally a lower level of antioxidant enzymes than RHE or skin explants. If we compare cells in monolayers, NHEK displays very low expressions of catalase and SOD1 as compared to HaCaT cell line. This must be considered for the choice of the most relevant model to assess the antioxidant activity or the ROS production, as examples. Besides, primary keratinocytes express a higher level of catalase and SOD transcripts when differentiated to form a 3D epidermis compared to NHEK grown in monolayer. On the contrary, they display a lower level of GPX than 2D cell culture models. These results suggest that the differentiation process in human epidermis affects the expression of antioxidant enzymes. Nevertheless, we cannot exclude that the presence of fibroblasts under the membrane in RHE plays also a role in controlling antioxidant enzyme expression in keratinocytes. This work confirms studies performed on catalase in animal models. In fact, in newborn rat keratinocytes, catalase was reported to be mainly expressed in the cytoplasm of cells inside the upper layers, the *stratum granulosum* and the *lower corneum* [19]. Besides, catalase-specific activity was shown to increase after cessation of cell division of murine primary keratinocyte in *in vitro* monolayer culture whereas it remains relatively constant during the proliferation phase [20]. Furthermore, in murine skin, the study of keratinocyte subpopulations in different stages of differentiation confirmed that catalase activity increases with keratinocyte maturity [21]. In the present work, we demonstrated that, in the human model, *in vitro* keratinocyte differentiation also controls catalase expression. In addition, we show that it also modulates SOD and GPX expressions.

Interestingly, RHE show a similar antioxidant profile as skin explants with an overexpression of catalase and SODs and a low expression of GPX family genes. This confirms that the RHE models closely mimic the real epidermis. So, *in vitro* epidermis differentiation process leads to the same antioxidant profile as *in vivo* and highlights the relevance of using RHE for skin antioxidant investigations. Comparatively, HaCaT and NHEK monolayer models are not representative of the antioxidant defences of the skin.

### 3.2. Characterization of Keratinocyte Proliferation in Physioxia Condition

**Figure 2:**
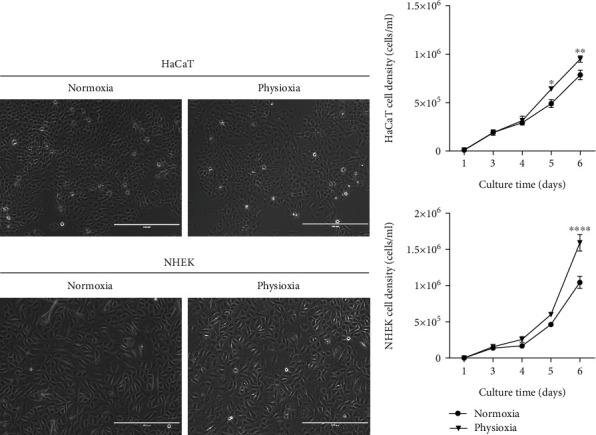
Differences in HaCaT and NHEK proliferation rates in physioxia vs. normoxia. HaCaT and NHEK were maintained either in physioxia (3% O_2_) or in normoxia (18.6% O_2_) for one week. (a) Phase contrast images of HaCaT and NHEK cultures at four days after seeding, scale bar: 400 *μ*m. (b) Proliferation curves of HaCaT and NHEK in physioxia and in normoxia. Cells were quantified using the NucleoCounter® allowing the evaluation of viable cells. Data are represented with mean ± SEM of 2 independent experiments including 6 replicates each. Statistical significance was determined using analysis of variance (ANOVA). ^∗^*p* < 0.05, ^∗∗^*p* < 0.01, ^∗∗∗^*p* < 0.001, and ^∗∗∗∗^*p* < 0.0001.

To characterize the effect of oxygen on keratinocytes, cell cultures were grown in physioxia conditions (3% oxygen) for four days, to mimic epidermis physiological conditions, and compared to culture in normoxia (18.6% oxygen). No morphological change was observed for HaCaT and NHEK in both oxygen conditions (Figure 2(a)). However, we show a significant increase in the cell proliferation rate in physioxia when compared to normoxia (+20% and +52% on day 6 after seeding, respectively, for HaCaT and for NHEK) (Figure 2(b)). Our results are consistent with the previous findings showing an increase in keratinocyte proliferation in physioxia (2 to 5% O_2_) after 4 to 6 days of culture [3, 5]. Particularly, freshly isolated keratinocytes grown for 8 days in the absence of serum and exposed to 5% of oxygen were shown to increase their growth rate up to 12-fold compared to the same cells grown in normoxia [22]. Interestingly, primary keratinocyte growth at 5% oxygen reduces differentiation gene expression like K10, involucrin, and filaggrin [22]. All these results support the idea that physioxia allows keeping the proliferative potential of keratinocyte and preserving them from differentiation. However, two studies reported a decrease in keratinocyte proliferation at 3 and 4% O_2_ for shorter terms of culture (2 to 4 days) [23, 24]. These conflicting reports show how the role of oxygen on keratinocyte growth is still not very well understood.

Besides, physioxia was also shown to increase cell proliferation of skin cell types such as melanocytes [25] and dermal papilla cells at 2% O_2_ [26] but displays a lack of effect on human skin fibroblast [27]. In addition, the importance of the right oxygen level for skin cell growth and differentiation was demonstrated in a study on hair follicles where growing conditions in normoxia resulted in the most efficient induction of dermal papilla cells, whereas low oxygen level caused the most efficient induction and maturation of dermal sheath cells [28]. Interestingly, several recent studies point in the same direction and highlight the importance of respecting physioxia in cell culture in order to keep the original cell phenotype. This was demonstrated for endothelial progenitor cells and adipocytes keeping their angiogenesis and their migratory abilities with a lower proportion of senescent cells [29, 30] and for chondrocytes retaining their chondrogenicity [31].

### 3.3. Characterization of RHE Generated in Physioxia

Keratinocyte monolayer cultures are considered as representative of proliferative keratinocytes, i.e., cells from the basal layer of the epidermis, but they do not integrate the differentiation process that occurs in the upper layers. Closer to skin physiology, RHE are now mainly used to study skin mechanisms as they reproduce a functional barrier. To study the effects of oxygen level, we generated RHE in physioxia and characterized their properties in comparison to normoxia.

**Figure 3:**
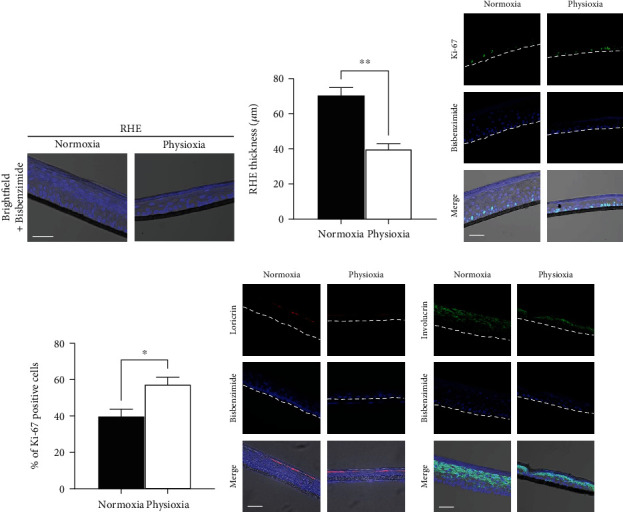
Comparison between RHE generated in physioxia and in normoxia. RHE was prepared in physioxia (3% O_2_) and in normoxia (18.6% O_2_). (a) Nucleus staining with bisbenzimide; (b) evaluation of epidermis thickness; (c) visualization of proliferative cells by Ki67 immunostaining; (d) evaluation of the percentage of Ki67-positive cells in the basal layer of RHE; (e) differentiation markers, involucrin and loricrin, immunostaining. Scale bar: 50 *μ*m. Data are represented with mean ± SEM of at least 3 independent experiments. Statistical significance was determined using analysis of variance (ANOVA). ^∗^*p* < 0.05 and ^∗∗^*p* < 0.01.

Following 15 days of culture, RHE were developed in both oxygen conditions. They display a correct cell stratification shown by the nucleus staining and the presence of a stratum corneum (Figure 3(a)). However, we observed structural and biological differences in epidermis formation in physioxia compared to RHE grown in standard conditions. Interestingly, a lower epidermis thickness was observed in physioxia with about 40 *μ*m compared to 70 *μ*m in normoxia (Figures 3(a) and 3(b)). This can be correlated to the increase in Ki67-positive cells (+43%, Figures 3(c) and 3(d)) showing that keratinocytes from the basal layer rather stay in the proliferation phase than enter the differentiation process, leading to a thinner epidermis. However, the differentiation markers, loricrin and involucrin, are present in physioxia (Figure 3(e)) in the upper layer under the stratum corneum and indicate an achieved epidermal terminal differentiation.

To our knowledge, very few studies focused on the role of oxygen on skin 3D models and its consequences. The stratification of keratinocytes in a colony formation model was reported to be greatly attenuated at 2% O_2_ [32]. Additionally, a low epidermis thickness was reported for RHE generated at 5% and 2% O_2_, and a delayed cornification was observed below 10% O_2_ [22]. The terminal differentiation that we observed in our results and that is absent from the previous model could be due to the presence of the fibroblasts under the membrane as they were shown to crosstalk with keratinocytes and enhance epidermal barrier formation [33]. This is in agreement with the second study on a full-skin model prepared at 3% O_2_ where a thinner epidermis was shown as well as a terminal differentiation with the expression of loricrin, involucrin, and filaggrin [24]. Of interest, the authors reported that, according to the study of the *stratum corneum* composition and the lipid organisation, full-skin equivalents prepared in physioxia were shown closer to the native human skin [24]. Taken together, these results suggest that the exposition of skin cells, which are naturally in physioxia, to increased levels of oxygen in *in vitro* cultures accelerates keratinocyte differentiation, leading to a thicker epidermis with less proliferating cells.

### 3.4. Physioxia Decreases H_2_O_2_-Induced Intracellular ROS Production in Keratinocytes

In the cellular metabolism, oxygen availability is closely related to the production of ROS through mitochondria and enzyme activities [34]. To understand the role of oxygen in the antioxidant defences of the skin, we started by evaluating the production of ROS in keratinocytes in physioxia and in normoxia, both in basal conditions and after induction of H_2_O_2_ stress.

**Figure 4:**
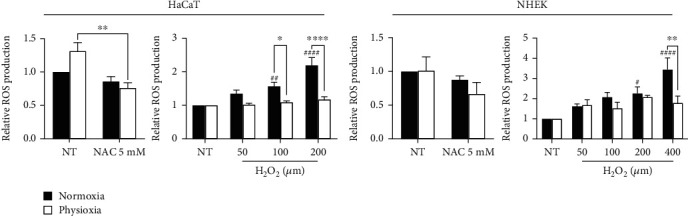
Decrease in H_2_O_2_-induced ROS production in keratinocytes under physioxia condition. HaCaT and NHEK, in physioxia or in normoxia, were submitted to H_2_O_2_ stress (50 to 400 *μ*M) for 20 min. ROS production was evaluated using the CM-H_2_DCFDA probe. Nontreated (NT) cells were incubated with N-acetyl-cysteine (NAC, 5 mM), a ROS scavenger. Basal ROS production was expressed as normalized values versus normoxia condition. H_2_O_2_-stimulated ROS production was expressed as normalized values versus the corresponding control, normoxia or physioxia. Data are represented with mean ± SEM of at least 3 independent experiments. Statistical significance was determined using analysis of variance (ANOVA). ^##^*p* < 0.01 and ^####^*p* < 0.0001 versus the corresponding control, physioxia or normoxia. ^∗^*p* < 0.05, ^∗∗^*p* < 0.01, ^∗∗∗^*p* < 0.001, and ^∗∗∗∗^*p* < 0.0001 between physioxia and normoxia.

In both HaCaT and NHEK, the basal production of ROS (Figure 4) is not significantly different between physioxia and normoxia after 4 days in culture, and only part of this production can be scavenged by NAC in HaCaT cells. Keratinocytes keep thus a stable ROS level despite the strong oxygen variation. In the literature, ROS production in cells at physiological oxygen level is still misunderstood, and the results are different from one study to another. Kato et al. show a decrease in primary human oral keratinocyte ROS at 2% O_2_ for 72 h [5]. This was also reported for human dermal fibroblasts at various O_2_ levels (1-10% O_2_) with a linear basal ROS decrease with the diminution of O_2_ [27] and for cells from dermal papillae at 2% O_2_ [26] or 5% O_2_ [7]. However, an increase in basal ROS production was reported in human adipocytes at 2% O_2_ [30] and similarly in human dermal fibroblasts, young or old, at 5% O2 but with a concomitant increase in intramitochondrial superoxide [8]. Therefore, the production of basal ROS in physioxia depends not only on the cell type or on the oxygen level but also maybe on the culture medium, or on the choice of the ROS probes.

Besides basal ROS production, we evaluated the ability of cells to respond to H_2_O_2_ stress in physioxia. Upon stimulation with high levels of H_2_O_2_, both HaCaT and NHEK show a significant increase in ROS production but only in normoxia (Figure 4). Interestingly, under physioxia condition, the effects of H_2_O_2_ were almost totally inhibited because the ROS generation at different H_2_O_2_ concentration was not significantly different than that in the control group, for both cellular types. The difference in ROS production between physioxia and normoxia is significant starting from 100 *μ*M for HaCaT and only at 400 *μ*M for NHEK. This result shows that cells in physioxia are less sensitive to H_2_O_2_ stress, even for high concentrations, and may suggest that they possess a better antioxidant system.

### 3.5. Physioxia Decreases Antioxidant Gene Expression in Keratinocyte Cultures and in RHE

**Figure 5:**
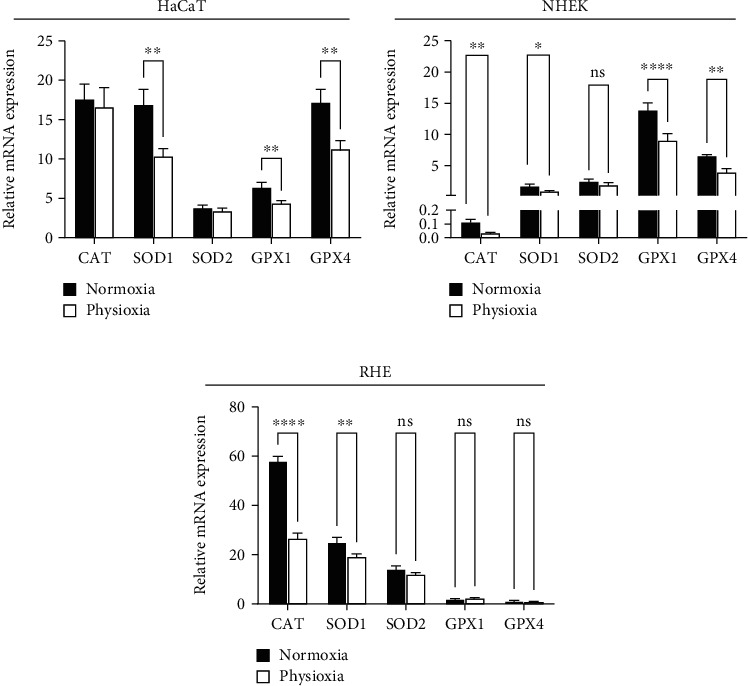
Physioxia decreases antioxidant gene expression in keratinocyte cultures, HaCaT (a), NHEK (b), and RHE (c). HaCaT and NHEK were grown in physioxia (3% O_2_) or normoxia (18.6% O_2_) for 4 days. RHE were generated in physioxia or in normoxia for 15 days. Quantitative real-time PCR analyses were performed for mRNA expression of catalase, SOD1, SOD2, GPX1, and GPX4. The results were normalized to the three housekeeping genes GAPDH, B2M, and GUSB. Data are represented by the relative mRNA expression with mean ± SEM of at least 3 independent experiments. Statistical significance was determined using analysis of variance (ANOVA). ^∗^*p* < 0.05, ^∗∗^*p* < 0.01, ^∗∗∗^*p* < 0.001, and ^∗∗∗∗^*p* < 0.0001 between physioxia and normoxia.

ROS are continually generated inside the cell in response to various endogenous and exogenous signals. Therefore, the expression and the activity of antioxidant enzymes are crucial for maintaining a controlled redox state and for preventing damages to biological molecules. As physioxia decreases ROS level upon H_2_O_2_ stress, we investigated the expression of the main antioxidant enzyme genes using RT-qPCR, comparing normoxia and physioxia conditions. In both HaCaT and NHEK, SOD1, GPX1, and GPX4 expressions were significantly downregulated at 3% O_2_ (Figures 5(a) and 5(b)). The decrease was of -38%, -31%, and -34%, respectively, for SOD1, GPX1, and GPX4 in HaCaT and -45%, -34%, and -39% in NHEK. On RHE (Figure 5(c)), the downregulation of SOD1 was confirmed (-23%) whereas the GPXs were not affected by physioxia. On both NHEK and RHE but not on HaCaT, catalase expression was strongly reduced (-68% and -54%, respectively) in physioxia. Besides, we demonstrated here that SOD2 transcript remains unchanged in physioxia whatever the model used.

The effect of oxygen level on antioxidant profile is still little studied, and up to now, nothing was reported for keratinocytes. A similar result about a decrease in SOD1 expression, and also in SOD3, was reported in dermal fibroblasts, whether young or old, but in these cells, the catalase expression remained unchanged [8]. In accordance with our findings and in a slightly more distant cell type, the A431 human epidermoid carcinoma, Ferguson et al. described the same phenotype in physioxia with a reduction of SOD1, GPX1, and catalase expressions and a lack of SOD2 modulation under culture at 2% O_2_ [35]. In contrary, mRNA of SOD1 was reported to be increased in several cell line cultured at 3% of oxygen [36].

### 3.6. Physioxia Regulates Antioxidant Enzyme Amounts in Keratinocyte Cultures and in RHE

As the decrease in antioxidant enzyme expression was not consistent with our hypothesis that cells in physioxia display a better antioxidant efficacy, we then investigated the antioxidant enzymes at the protein level by immunofluorescence and Western blotting.

**Figure 6:**
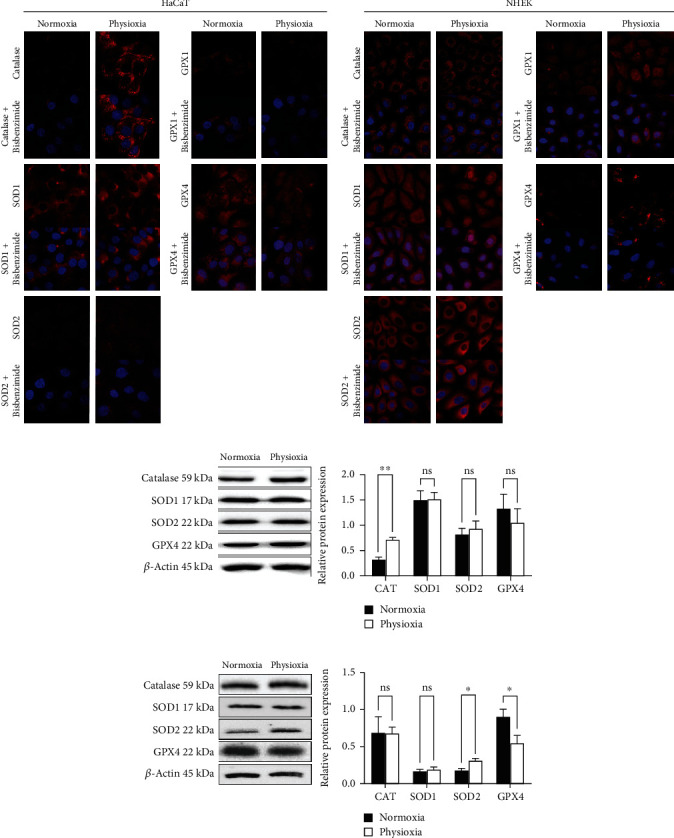
Physioxia modulates antioxidant enzyme amounts in keratinocyte cultures. HaCaT and NHEK were submitted to physioxia (3% O_2_) or normoxia (18.6% O_2_) for 4 days. The catalase, SOD1, SOD2, GPX1, and GPX4 were visualized by red immunofluorescence with a counterstain of nuclei with bisbenzimide in HaCaT (a) and in NHEK (b). Scale bar: 40 *μ*m. Western blotting pictures and quantification of catalase, SOD1, SOD2, and GPX4 in HaCaT (c) and in NHEK (d). Data are represented by the relative protein amount with mean ± SEM of at least 3 independent experiments. Statistical significance was determined using analysis of variance (ANOVA). ^∗^*p* < 0.05 and ^∗∗^*p* < 0.01, between physioxia and normoxia.

**Figure 7:**
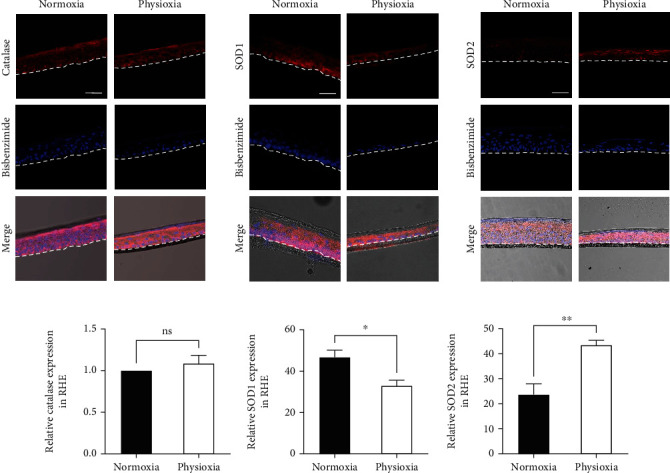
Physioxia modulates antioxidant enzyme amounts in RHE. RHE were generated in physioxia (3% O_2_) or in normoxia (18.6% O_2_) for 15 days. (a) Catalase, SOD1, and SOD2 were visualized by red immunofluorescence with a counterstain of nuclei with bisbenzimide. Scale bar: 50 *μ*m. (b) Quantification of catalase, SOD1, and SOD2 from immunostainings. Data are represented by the relative protein amount with mean ± SEM of at least 3 independent experiments. Statistical significance was determined using analysis of variance (ANOVA). ^∗^*p* < 0.05 and ^∗∗^*p* < 0.01, between physioxia and normoxia.

In HaCaT, a clear increase in catalase amount in the cytoplasm in physioxia was observed by immunofluorescence, and this was confirmed by the quantification of Western blotting (+121% in physioxia compared to normoxia) (Figures 6(a) and 6(c)), showing a lack of correlation with the mRNA content. However, the amount of catalase remained unchanged in NHEK (Figures 6(b) and 6(d)) and in RHE (Figure 7). Taken together, these results show an increase in catalase without modulation of the corresponding mRNA in HaCaT and a lack of catalase modulation associated with a downregulation of the mRNA in NHEK and RHE. By contrast, a decrease in catalase amount was previously reported in dermal fibroblasts at 1% O_2_ [27] and in dermal papilla cells at 2% O_2_ [26], but in these two studies, the corresponding gene expressions were not assessed. Concerning SOD2, we found in NHEK that the protein amount increases in the cell cytoplasm in physioxia with a significant level (+82%). Similarly, SOD2 amount is higher (+86%) in RHE in physioxia, confirming the result obtained in NHEK.

For SOD1, we demonstrated a reduction in physioxia of protein amount in RHE (-30%) whereas it was unchanged in keratinocyte monolayer cultures. However, we observed a preferential accumulation of SOD1 inside the nuclei of NHEK in physioxia compared to normoxia whereas in HaCaT, it remains in the cytoplasm (Figures 6(a) and 6(b)). Interestingly, it was previously demonstrated that SOD1 can be present in the nucleus of fibroblasts where it can act as a transcription factor. This intranuclear localization was shown to be triggered by the presence of high levels of H_2_O_2_, and therefore, SOD1 regulates oxidative resistance and repair gene expression [37]. Whether a higher production of H_2_O_2_ is present in NHEK in physioxia, compared to normoxia, despite an unchanged global ROS production (Figure 4) remains to be determined. In addition, the different localizations of SOD1 we observed in physioxia in NHEK compared to HaCaT might be explained by a different content in H_2_O_2_. This is consistent with our findings showing the higher catalase gene expression in HaCaT compared with NHEK.

Confirming partly our results, a study of keratinocytes from either old or young patient at 4% O_2_ reported a lack of SOD1 modulation, but for SOD2 also [23] whereas another study on dermal fibroblasts at 1% O_2_ showed a decrease in both enzyme amounts [27].

A questionable point in this study of antioxidant enzymes is the decrease, or stability, in mRNA expression, both in 2D and in 3D models, correlated with an increase of protein amount, catalase in HaCaT and SOD2 in NHEK and RHE. Several hypotheses can be raised such as a higher protein stability in physioxia or a change in enzyme translation mechanisms. In fact, Timpano and Uniacke demonstrated that human cells cultured under physiological oxygen (1-8% O_2_) utilize two cap-binding proteins to recruit distinct mRNAs for translation, eIF4E dominant in normoxia, and eIF4E2 dominant in hypoxia, whereas only eIF4E-based translation is active in normoxia [38]. For each cap-binding protein being specific for different mRNA [39], this may explain that, even if the mRNA expression is low in physioxia, the protein can be more expressed if eIF4E2 is more specific for this mRNA than eIF4E in physioxia condition. These hypotheses remain to be further explored.

### 3.7. Physioxia Stimulates Antioxidant Enzyme Activity in Keratinocytes

**Figure 8:**
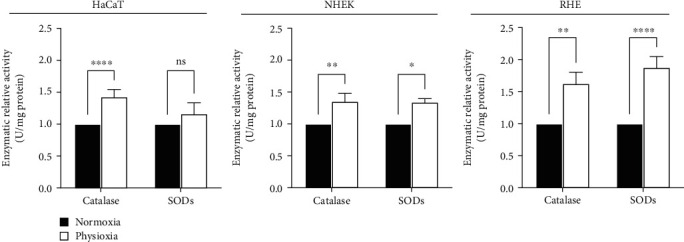
Physioxia stimulates antioxidant enzyme activity in keratinocyte cultures and in RHE. HaCaT and NHEK were submitted to physioxia (3% O_2_) or normoxia (18.6% O_2_) for 4 days. RHE were generated in physioxia (3% O_2_) or in normoxia (18.6% O_2_) for 15 days. Catalase and SOD enzymatic activities were evaluated in cells or RHE extracts. Data are presented by the relative enzymatic activities, normalized to protein amount, with mean ± SEM of at least 3 independent experiments. Statistical significance was determined using analysis of variance (ANOVA). ^∗^*p* < 0.05, ^∗∗^*p* < 0.01, ^∗∗∗^*p* < 0.001, and ^∗∗∗∗^*p* < 0.0001 between physioxia and normoxia.

As the presence of the enzymes in terms of protein amount does not predict enzyme activity, we further assessed the antioxidant activity of catalase and SODs in the same culture models (Figure 8). The results show a similar and significant increase in catalase activity in physioxia for HaCaT, NHEK, and RHE (+41%, +35%, and +62%, respectively). Additionally, the superoxide dismutase activity was higher in physioxia in NHEK (+33%) and much higher in RHE (+87%) but was unchanged in HaCaT.

Although keratinocytes are the richest cells in antioxidant enzymes in the skin relative to the dermal cells [14], almost nothing is known about the effect of oxygen level on antioxidant activity. However, a lower antioxidant activity has been reported in physioxia in other cell types. A decrease in catalase activity was described in cells from the dermal papilla [26]. Also, a decline in the expression of antioxidant enzymes and their activities was observed in carcinoma cells cultivated at 2% O_2_ [35], as well as in fibroblasts at 1% O_2_ [27]. These results are not consistent with ours, obtained in keratinocytes, and up to now, no mechanism has been put forward to explain such effect.

The higher SOD activity in physioxia can be explained by our work showing an increase in SOD2 protein amount (Figure 6). However, catalase seems to be affected differently because its activity is not correlated to the protein level. Our findings suggest that catalase becomes overactivated in physioxia in both NHEK and RHE. At least two mechanisms have been reported in the literature able to modulate catalase activity. Some posttranslational changes in catalase have been identified that may influence a conformational change in the enzyme and thus its activation or inhibition. The glycation onto specific catalase lysine residues, for example, can lead to its activation [40]. Catalase activity was also shown to be modulated by direct interactions with other proteins, p53R, a transcriptional product of p53 [41], or calmodulin, the calcium regulation protein, a mechanism identified in plant [42], but not yet in mammalian cells. As calmodulin is present in the upper layers of the epidermis [43], a difference in its concentration or in its localization in physioxia may increase catalase activity. These hypotheses may explain why catalase activity can be induced independently of its protein amount, but their relationship with the oxygen level has now to be investigated.

Our results show here the same increase of antioxidant activities in physioxia versus normoxia in the three keratinocyte models, with a higher effect in RHE, except for SOD in HaCaT cell line where the increase is not significant. However, our work reveals that the three models differ in their basal antioxidant profile and also in their response to physioxia in terms of gene and protein expressions or localization, mainly between the HaCaT cell line and NHEK. As the HaCaT cell line has been grown for a long time in atmospheric culture conditions, we can hypothesize that this conditioning induced a stable overexpression of catalase and SOD1 compared to primary keratinocytes, NHEK, and that it may alter their regulation by oxygen level changes. In fact, physioxia only increases catalase expression and activity without effect on SOD.

Besides, NHEK and RHE displayed quite similar modulations in physioxia even if their basal antioxidant profile is very different, likely related to keratinocyte differentiation. Therefore, the present work points out the importance of the oxygen level but also of the keratinocyte model choice to represent skin physiological conditions in *in vitro* experiments.

## 4. Conclusion

In this study, we successfully established various models of keratinocyte cultures in physioxia, in particular, reconstructed epidermis, and demonstrated that physiological oxygen conditions controlled both keratinocyte proliferation and the expression of antioxidant enzymes. As a consequence, a better protection of keratinocytes against oxidative stress was found under physioxia conditions compared to classical cultures in normoxia, mainly due to the overproduction of SOD2, and to the overactivation of catalase, in NHEK and RHE. Further studies are needed to elucidate the mechanisms underlying such modulations of antioxidant enzymes by oxygen level. This work contributes to the understanding of the role of oxygen in the regulation of skin functions and highlights the importance to respect skin physioxia. Furthermore, the antioxidant system being highly involved in the prevention of the skin accelerated aging mainly due to exposome, our findings bring a new *in vitro* model to study skin aging in conditions closer to skin physiology.

## Data Availability

All the data used to support the findings of this study are available from the corresponding author upon request.

## Abbreviations

B2M: Beta-2 microglobulin
CM-H_2_DCFDA: 5-(and-6)-Chloromethyl-2′,7′-dichlorodihydrofluorescein diacetate
DMEM: Dulbecco's Modified Eagle's Medium
GAPDH: Glyceraldehyde-3-phosphate dehydrogenase
GPX: Glutathione peroxidase
GUSB: *β*-Glucuronidase
HDF: Human dermal fibroblast
NHEK: Normal human epidermal keratinocytes
PBS: Phosphate-buffered saline
RHE: Reconstructed human epidermis
ROS: Reactive oxygen species
SOD: Superoxide dismutase
TBS: Tris-buffered saline.
